# First-into-man phase I and pharmacokinetic study of XR5944.14, a novel agent with a unique mechanism of action

**DOI:** 10.1038/sj.bjc.6603953

**Published:** 2007-09-11

**Authors:** W Verborg, H Thomas, D Bissett, J Waterfall, J Steiner, M Cooper, E M Rankin

**Affiliations:** 1 Division of Cancer Medicine, University of Dundee, Ninewells Hospital and Medical School, Dundee DD1 9SY, UK; 2 St Luke's Cancer Centre, The Royal Surrey County Hospital, Egerton Road, Guildford GU2 5XX, UK; 3 Grampian Universities NHS Trust, Foresterhill, Aberdeen AB25 2XG, UK; 4 Xenova Ltd, 957 Buckingham Avenue, Slough, Berkshire SL1 4NL, UK; 5 Oxford Therapeutics Consulting Ltd, Brightwell cum Sotwell, Wallingford, Oxford OX10 1BB, UK; 6 Millennium Pharmaceuticals, Inc, 35 Landsdowne Street, Cambridge, MA 02139, USA

**Keywords:** XR5944.14, phase I, solid tumours

## Abstract

The bis-phenazine XR5944.14 is a novel cytotoxic agent which intercalates into DNA and inhibits transcription. The objectives of this study were to determine the dose-limiting toxicity (DLT), the maximum tolerated dose (MTD) and to describe the pharmacokinetics (PKs) of XR5944.14 when given at doses ranging from 3.6 to 36 mg m^−2^ every 3 weeks to patients with advanced tumours. Twenty-seven patients were treated with a total of 77 cycles. Dose-limiting toxicities occurred at doses ⩾24 mg m^−2^. Oral mucositis was the most common DLT. Two patients developed acute renal failure possibly related to the study drug. Other less-severe toxicities were diarrhoea, nausea, vomiting and fatigue. Haematological toxicity was mild. One patient showed an objective partial response. Pharmacokinetic analysis was performed during the first cycle of treatment and plasma was assayed for XR5944.14 using a validated liquid chromatography tandem mass spectrometry. The systemic exposure of XR5944.14 increased more than proportionally with increasing dose, with inter-patient variability increasing from dose level 24 mg m^−2^ onwards. The lack of correlation between toxicity and PK values makes it difficult to recommend a dose for further study in phase 2 trials. More work is needed to explain the inter- and intra-individual variation in PKs and pharmacodynamics.

Molecularly targeted therapies offer much hope for selected tumours. However, cytotoxic compounds are still the mainstay of systemic therapy for most malignancies. There remains a need for agents with novel mechanisms of action which offer a broad spectrum of antitumour activity and an improved safety profile compared with existing anticancer agents.

The bis-phenazine XR5944.14 (MLN 944) is a novel cytotoxic agent (Figure 1). Initially, it was thought to interfere with the normal function of topoisomerase I and II (Stewart *et al*, 2001). Recent studies, however, have indicated that the agent intercalates into DNA and inhibits transcription, but has no effect on the catalytic activity of either topoisomerase I or II (Dai *et al*, 2004; Byers *et al*, 2005). XR5944.14 induces a G(1) and G(2) cell cycle arrest (Sappal *et al*, 2004). Agents with this mechanism of action offer great promise because of their activity against a broad spectrum of tumour types (Di Nicolantonio *et al*, 2004).

The efficacy of XR5944.14 is not schedule-dependent, since the drug is able to kill tumour cells following only a brief exposure. In studies using murine and human tumour cell lines or xenograft models, XR5944.14 was exceptionally potent, showing several-fold greater activity than many common cytotoxic agents such as doxorubicin, camptothecin, topotecan or paclitaxel and synergy with carboplatin or doxorubicin (Harris *et al*, 2005a, 2005b). In nude mice bearing xenografts of human H69/P SCLC, both partial and complete regression of large established tumours were seen. Complete tumour regression was seen at well-tolerated doses of XR5944. XR5944 even delayed tumour growth in the HT29 human colon carcinoma, which is relatively refractory to cytotoxic chemotherapy (Stewart *et al*, 2001).

Preclinical studies in mice and rats showed that XR5944.14 produced bone marrow suppression and gastrointestinal epithelial damage. These effects were reversible and recovery was such that the drug could be given on a 3-weekly cycle. In dogs, prolongation of the QT-interval, acute urticaria and wheezing were seen, but this species is particularly sensitive to agents with the potential to cause histamine release.

In mice, following a single injection of 20 mg kg^−1^ (60 mg m^−2^), the plasma concentration *vs* time curve was biphasic, with a rapid decrease in the plasma concentration over the first 10 h, followed by a slower phase of plasma drug elimination with an estimated half-life of 21 h. Pharmacokinetic (PK) studies confirmed that XR5944.14 has a long elimination half-life and a large volume of distribution (22.8 l kg^−1^), which is consistent with the high plasma protein binding of XR5994. At 168 h after dosing [^14^C]XR5944.14 in the rat, 70% of the radioactivity was found in the faeces, 10% appeared in the urine and 20% was in the carcass.

There was a linear relationship between dose and systemic exposure to XR5944.14. The minimal effect dose of 0.6 mg kg^−1^ in rats was associated with a mean maximum plasma concentration (*C*_max_) of 21.7 and 13.8ng ml^−1^ on days 1 and 22, respectively. In dogs, the NOEL of 0.2 mg kg^−1^ (4 mg m^−2^) was associated with a mean *C*_max_ of 15.7 and 23.8 ng ml^−1^ on days 1 and 22, respectively.

The objectives of this phase I study were to determine the toxicity and the MTD of XR5944 when given as a 30-min infusion every 3–4 weeks and to describe the PKs of the agent. In addition, we wanted to document any antitumour activity and to establish the appropriate dose for phase II studies.

## PATIENTS AND METHODS

### Patient selection

Eligible patients had histologically confirmed, advanced solid tumours refractory to standard therapies. They met the following criteria: aged 18 years or more, ECOG performance status 0 or 1, expected survival longer than 3 months, recovered from the reversible effects of prior therapy, with at least 4 weeks since the last exposure to chemotherapy or radiotherapy and at least 6 weeks since exposure to nitrosoureas, mitomycin C or antibody therapy, no more than four previous cytotoxic chemotherapeutic regimens (including regimens used as adjuvant or neo-adjuvant therapies), clinically or radiologically evaluable tumours, adequate haematopoietic (neutrophil count ⩾1.5 × 10^9^ l^−1^, platelet count ⩾100 × 10^9^ l^−1^, renal (serum creatinine ⩽0.14 mmol l^−1^) and hepatic function (bilirubin ⩽1.5 the upper limit of normal (ULN), aspartate (AST) or alanine transferase (ALT) or alkaline phosphatase ⩽ twice the ULN). In patients with disease metastatic to the liver or bone, levels of AST and ALP up to five times the ULN were allowed. Patients were excluded if they had clinically significant abnormalities or arrhythmia on 12-lead ECG; had a QT_c_ of more than 450 ms on ECG; had clinical or radiographic evidence of cerebral metastases; had previous radiotherapy involving ⩾25% of haematopoietically active bone marrow; had previous high-dose chemotherapy requiring peripheral blood or bone marrow stem cell support; if they were taking drugs known to prolong QT interval. All patients gave written informed consent. The study was approved by MREC and the institutions' Local Research Ethics Committees.

### Treatment and dose escalation

**Figure 2:**
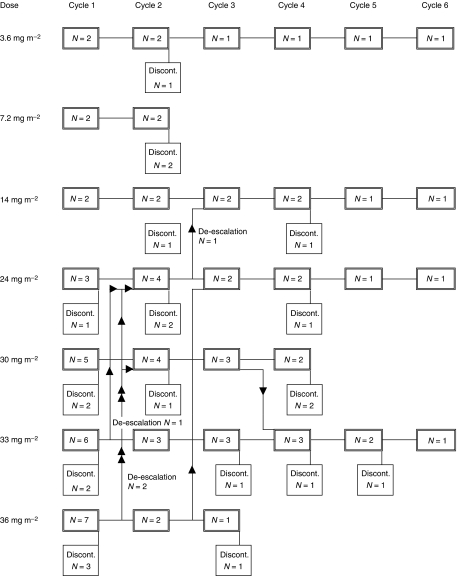
Patient disposition.

XR 5944.14. was supplied by Xenova Ltd as a freeze-dried vial containing XR5944.14 dimesylate (21.5 mg XR5944.14 free base per vial) and sodium hydroxide/hydrochloric acid. Each vial was reconstituted with water for injection to give a solution equivalent to 4 mg ml^−1^ XR5944.14 and the required dose was then added to a 250-ml (later 500 ml) bag of 5% glucose injection BP, which was stored for up to 4 h at 2–8°C before use. The drug was given over 30 min using a calibrated infusion pump and a PVC giving set fitted with an in-line filter. The infusion was repeated every 3 weeks, with a 1-week delay permissible to allow recovery from toxicity. A total of six cycles could be given. The starting dose was 3.6 mg m^−2^, which was less than 1/10 of the maximum tolerated dose (MTD) in the rat, and which represented a no observable adverse effect level in the dog. Dose escalation was based on the toxicity in the first cycle and followed a standard modified Fibonacci numerical scheme (Figure 2). Provision was made in the protocol for intermediate dose levels or expansion of a dose cohort to define better the dose–toxicity relationship of XR5944.14. If a patient experienced dose-limiting toxicity (DLT), the dose of XR5944 was reduced by at least one dose level at retreatment. Once dose reduction was required, no dose escalation was permitted. Blood and urine samples were collected for PK evaluation from each patient following the first infusion of XR5944.14.

Two patients were entered at each dose level until the occurrence of either two or more instances of common toxicity criteria (CTC) (Cancer Therapy Evaluation Programme, 1999) grade 2 toxicity in which case the cohort was expanded to three patients, or any CTC grade 3 or higher toxicity, in which case the cohort was expanded to include up to six patients. If DLT was observed in 1/3 patients treated at a given dose level, then a further three patients were added to that dose level. If none of these additional patients experience DLT (DLT in only 1/6 patients), then dose escalation resumed. If ⩾2 patients experienced DLT at a given dose level, then dose escalation ended. Dose-limiting toxicities were defined as: grade 4 neutropenia lasting for more than 7 consecutive days; febrile neutropenia; platelets <25 × 10^9^ l^−1^; any grade 3 or greater non-haematological toxicity (except injection site reactions, arthralgias/myalgias, alopecia, brief fatigue); QT_c_ prolongation >500 ms or clinically significant arrhythmia during the 24 h after infusion of XR5944.14; interval of more than 35 days between infusions because of toxicities. Second and subsequent cycles were given providing therapy-related toxicity had resolved to CTC grade 1 or less, neutrophil count was ⩾1.5 × 10^9^ l^−1^, platelets ⩾100 × 10^9^ l^−1^ and underlying tumour was stable or had improved.

### Treatment assessment

All patients at each dose level were observed for at least 48 h following the first infusion of XR5944.14. They were seen on days 4, 5 and 6 to evaluate possible toxic effects (graded according to the Common Toxicity Criteria version 2.0) and for further blood samples and measurement of vital signs. Repeat physical examination, blood (haematology and chemistry) and urine samples were done 24 h after the infusion and thereafter weekly. Computerised tomography (CT) scan of the relevant area was done pretreatment and every two cycles and tumour evaluated using the RECIST criteria (Therasse *et al*, 2000). Twelve-lead ECG (before and 15 min, 2, 6 and 24 h after the infusion) and 24-h ECG recording was done after each infusion.

### Pharmacokinetic blood and urine sampling

For PK analysis, 5 ml blood was drawn from the contralateral arm to the one used for the drug infusion. The samples were taken before and 10, 20, 30, 40, 50, 60, 75, 90, 120 min, 3, 4, 6, 8, 10, 24, 48, 72, 96 and 120 h after the first treatment. Blood samples were transferred to precooled polystyrene (Teklab, Sacriston, County Durham, UK) tubes containing lithium heparin then centrifuged at 2500 r.p.m for 10 min at 4°C within 15 min of collection. Plasma was stored frozen at −20°C until analysis.

Urine samples for PK analysis were taken 0–2, 2–4 and 8–24 h after the first infusion. Two 20 ml urine samples were drawn off and stored at −20°C until analysis. Levels of XR5944.14 were measured by a validated liquid chromatography tandem mass spectrometry at Huntingdon Life Sciences, Cambridgeshire.

### Pharmacokinetic data analysis

Actual times at which samples were taken were used throughout. Values that were below the lower limit of quantification (<0.1 ng ml^−1^) were entered as 0 in the calculation of the mean. Maximum plasma concentrations (*C*_max_) and their time of occurrence (*T*_max_) were the observed (measured) values. Areas under the plasma concentration–time curves up to 8 and 24 h post-dose (AUC_0–8_ and AUC_0–24_, respectively) and up to the last quantifiable sampling time (AUC_0–*t*_), were calculated using the logarithmic trapezoidal rule for declining concentrations and the linear trapezoidal rule when concentrations increased. The area under the plasma concentration–time curve extrapolated to infinity (AUC_0–∞_) was calculated as (AUC_0–*t*_+*C*_*z*_/*λ*_*z*_), where *C*_z_ is the observed plasma concentration at the time of the last quantifiable sample and *λ*_*z*_ is the terminal rate constant. Terminal rate constants were estimated by fitting a linear regression of log concentration against time. Terminal half-lives (*t*_½_) were calculated as ln 2/*λ*_*z*_. Total plasma clearance (CL) was calculated as Dose/AUC_0–∞_. Volume of distribution during the terminal phase (*V*_*z*_) was calculated as Dose/(*λ*_*z*_ × AUC_0–∞_) and volume of distribution at steady state (*V*_ss_) was calculated as CL × MRT, where MRT (mean residence time)=((AUMC_0–∞_/AUC_0–∞)_)−(duration of infusion/2)). The amount of XR5944.14 excreted unchanged in urine up to 48 h post-dose (Ae_0–48_) was calculated from the urine volume and concentration and by summing the amount excreted in each urine collection. The fraction of the dose excreted unchanged was calculated as Dose/Ae, and expressed as a percentage. Renal clearance (CL_R_) was calculated as Ae_0–48_/AUC_0–48_, where AUC_0–48_ is the area under plasma concentration–time curve up to 48 h post-dose. The AUC_0–48_ was calculated from data generated during this study using WinNonlin Pro, version 3.3 (Pharsight Corporation, Mountain View, CA, USA).

## RESULTS

Twenty-seven patients (11 male, 16 female, all Caucasians) with a median age of 61 (32–79) years were entered from three centres. All had had previous chemotherapy and nine patients had had radiotherapy. Two patients each entered the three lowest dose groups (3.6, 7.2 and 14 mg m^−2^), and three, five, six and seven patients were treated with 24, 30, 33 and 36 mg m^−2^ XR5944.14, respectively.

In five patients (19%), the dose was de-escalated and patients were treated further at a lower dose. No intra-patient dose escalation was planned but one patient received three cycles in the 30 mg m^−2^ dose group, and after discussion, the dose was escalated to 33 mg m^−2^ for cycle 4 again without toxicity but he developed progressive disease and stopped treatment (Figure 2). Four patients received the maximum of six cycles. The main reason for stopping (affecting 13 patients, 57%) was disease progression. Three patients (13%) discontinued due to a serious adverse event and three died of progressive disease. Two patients withdrew at their own request, one patient discontinued at the discretion of the investigator, and one more patient discontinued due to an adverse event. Six patients died within 30 days of treatment, four of progressive disease, one due to subarachnoid haemorrhage unrelated to XR5944.14 and one with renal failure possibly related to treatment (see below).

Two patients received cycle 1 at 24 mg m^−2^, without significant toxicity, so the next patient was treated with 36 mg m^−2^, but he experienced DLT. The next patient was treated with 24 mg m^−2^ without toxicity making a total of three patients treated at 24 mg m^−2^ without problems. An intermediate dose of 30 mg m^−2^ was introduced and three patients received this without DLT. The dose was then escalated back to 36 mg m^−2^ for a further four patients. One of these experienced DLT so two of the five patients given 36 mg m^−2^ had documented DLT. A further intermediate dose of 33 mg m^−2^ was introduced. Eight patients were enrolled at this dose level. Six patients in fact received 33 mg m^−2^, one of whom experienced DLT. Analysis of the data after the end of the study showed one patient received in error 37 rather than 33 mg m^−2^ but without DLT. One patient (patient 9) in fact received 39 mg m^−2^ rather than 33 mg m^−2^ because of an error in calculation of body surface area and he had DLT. These two patients have been allocated to the 36 mg m^−2^ dose level for the analysis of toxicity as this dose most closely approximates the actual dose given to these patients. While the study was ongoing however, it appeared that two of the eight patients allocated 33 mg m^−2^ dose experienced DLT in cycle 1. The dose was then de-escalated and the next two patients were enrolled at 30 mg m^−2^; both experienced DLT at which point the study was stopped. Dose-limiting toxicities were seen in nine patients altogether: in cycle 1, 30 mg m^−2^ – two patients, 33 mg m^−2^ – one patient, 36 mg m^−2^ – two patients; and in cycle 2, 24 mg m^−2^ – one patient, 30 mg m^−2^ – one patient, 36 mg m^−2^ – one patient; and one patient experienced DLT at 36 mg m^−2^ in cycle 1 and at 24 mg m^−2^ in cycle 2.

### Safety analysis

Twenty-seven patients were treated with a total of 77 cycles. Only toxicities occurring in the first cycle of treatment were considered in the decision to escalate the dose or expand the number of patients treated at a given dose level. The highest number of cycles (18) was given in the 33 mg m^−2^ group. A total of 12 DLTs were documented in nine patients, with oral mucositis being most common (Table 1). Grade 2 toxicity was dose-limiting if the patient failed to recover to grade 1 or less by day 28. The most common toxicities encountered were diarrhoea (15 patients), followed by oral mucositis (14 patients), nausea (13 patients), vomiting (12 patients) and fatigue (12 patients) all of which were more common at the higher doses (Table 2). Neutropenia was the only toxicity showing a trend with increasing dose (Table 2). Oral mucositis was the toxicity most often leading to a serious adverse event (five patients), followed by diarrhoea (three patients). Three patients developed renal failure. Nine days after 30 mg m^−2^, patient 3 developed fever and grade 2 mucositis and diarrhoea. He was admitted the following day, rehydrated with intravenous fluids and within 24 h was apyrexial after tazocin and gentamicin. However, the serum creatinine had risen to 290 *μ*mol. He was given haemodialysis for 4 days then treatment was stopped at his request. He died on day 19 with progressive metastatic colon cancer and renal failure. The relative contribution of XR5944.14 and gentamicin to the renal failure are unclear. The AUC_0–24_ was 1450 ng h ml^−1^ and the *C*_max_ was 1782 ng ml^−1^. Patient 4 had a serum creatinine of 243 *μ*mol 2 days after 30 mg m^−2^. Investigations showed obstructive uropathy. Attempts at ureteric stenting failed and the patient refused nephrostomies, dying of progressive pulmonary disease on day 30. Patient 9 was on ibuprofen and, like patient 3, had a normal serum creatinine at study entry. After 36 mg m^−2^ (in reality 39 mg m^−2^), the AUC_0–24_ was 917 ng h ml^−1^ and *C*_max_ 1070 ng ml^−1^. This patient developed acute renal failure associated with dehydration related to severe mucositis, diarrhoea and colitis that responded to antibiotics and steroids and he recovered. One patient developed a severe injection site reaction, which took several weeks to heal and this patient withdrew from the study. Due to the unpredictable nature of toxicities, no MTD could be identified.

Overall, the haematological toxicity was mild (Table 2). One patient in the 36 mg m^−2^ group had neutropenic sepsis. The patient recovered and the dose was reduced from 36 mg m^−2^ to 24 mg m^−2^ without recurrence. There was no evidence of cumulative toxicity. The evaluation of ECG or echocardiogram data did not raise specific safety concerns for XR5944.14 treatment.

### Efficacy

One patient developed a partial response according to RECIST criteria. This 53-year-old patient had progressed during fourth-line treatment for advanced ovarian cancer before being entered into the trial. The CT scan after cycle 2 showed a partial response according to the RECIST criteria. Before the CT scan could be repeated after 1 month to confirm the response the patient died. The precise cause of death was unclear. No patients showed a complete response. None of the patients showed improvements in performance status in the course of the study.

### Pharmacokinetics

**Figure 3:**
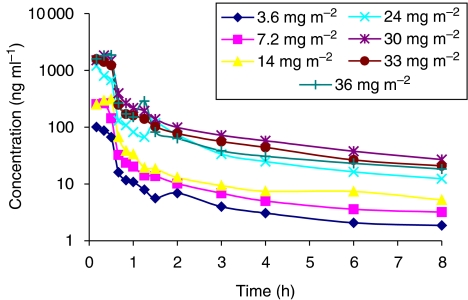
Mean plasma concentrations of XR5944.14 following a single intravenous infusion of XR5944.14 (log-linear plot 0–8 h).

**Figure 4:**
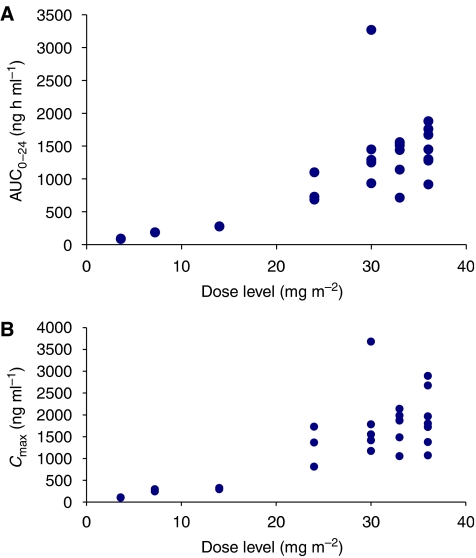
(**A**) Relationship between AUC_0–*t*_ and dose level following a single intravenous infusion of XR5944.14. (**B**) Relationship between *C*_max_ and dose level following a single intravenous infusion of XR5944.14.

Plasma concentration–time profiles of XR5944.14 up to 120 h post-dose were similar for all patients regardless of dose and are shown in Figure 3. Mean values of the PK parameters are presented in Table 3. The *C*_max_, AUC_0–*t*_ and AUC_0–∞_ values increased with increasing dose, and over the dose range 3.6–14 mg m^−2^, these increases appeared to be approximately proportionate to the dose increment. However, over the dose range 24–36 mg m^−2^, the increases were greater than the proportionate dose increment. Overall, the *C*_max_ and AUC_0–*t*_ values at the highest dose level (36 mg m^−2^) were 1.8-fold higher than those values predicted from a linear relationship, and there was statistically significant evidence of non-proportionality (Table 4). Inspection of the scatterplots of dose *vs* either AUC_0–24_ (Figure 4A) or *C*_max_ (Figure 4B) for XR5944.14 revealed an increase of both parameters with the dose level administered. The inter-patient variability increased from dose level 24 mg m^−2^ onwards. Mean *C*_max_ and AUC_0–24_ were similar in the higher dose levels. This might explain the similar toxicity profile seen in the higher dose groups (from 24 mg m^−2^ onwards). The *t*_½_, CL (clearance), MRT, apparent volume of distribution at steady state (*V*_ss_) and apparent volume of distribution during the terminal phase (*V*_*z*_) tended to decrease with increasing dose, indicating that the kinetics of XR5944.14 appeared to be nonlinear, and there was statistically significant evidence of nonlinearity for CL, *V*_*z*_ and *V*_ss_ (Table 3). The decrease in CL with increasing dose may indicate that metabolism of XR5944.14 is saturable, and the decrease in the volume of distribution may indicate saturation of tissue binding at higher dose levels.

The urinary excretion of unchanged XR5944.14 was low and in the range of 0.65–3% of the administered dose, increasing with the dose over the range 3.6–36 mg m^−2^. The low urinary excretion of unchanged XR5944.14 indicates that it is mainly excreted via another route and suggests that XR5944.14 may undergo extensive metabolism. There was no correlation between *C*_max_ or AUC_0–∞_ and the occurrence or grade of toxicities (Table 1).

## DISCUSSION

This open label, dose-escalation phase I trial describes the first administration of XR5944. 14 to man. The primary objectives of the study were to determine the toxicity and the MTD of XR5944.14 when administered as a 30-min infusion once every 3 to 4 weeks. The most frequently observed toxicities were diarrhoea, mucositis, nausea and vomiting. Preclinical toxicologic examination of XR5944.14 had indicated that cardiac enlargement, prolongation of QT_c_ intervals, allergic reactions and adverse effects on hepatic function might be expected and patients were monitored closely for these events. However, an evaluation of the respective toxicities and laboratory data did not raise specific safety concerns. Renal function was closely monitored in the study due to preclinical toxicological findings. No increase in proteinuria was observed in the study. Renal laboratory variables seemed more likely to deteriorate in the course of the study than other clinical chemistry variables, but several of these patients had progressive pelvic disease potentially contributing to renal dysfunction. Three patients developed acute renal failure, two of whom had concomitant mucositis (grades 2 and 3, respectively) and diarrhoea (grades 2 and 3, respectively) causing dehydration and in one, gentamicin may have contributed to renal problems. However, close monitoring for renal function in future administration of XR5944.14 to man is warranted.

The lowest dose associated with significant toxicity was 24 mg m^−2^ and DLTs were observed also in the 30, 33 and 36 mg m^−2^ dose groups. The maximum dose that could be given was 36 mg m^−2^. Although intermediate doses of 30 and 33 mg m^−2^ were explored, no clear MTD could be identified as the toxicity patterns proved unpredictable. Half of all DLTs and most dose de-escalations (three out of five) occurred in the 36 mg m^−2^ dose group. Between 30 and 36 mg m^−2^, an increase in the occurrence of toxicities was observed, suggesting doses <36 mg m^−2^ for potential future studies. Dose *vs* either AUC_0–*t*_ or *C*_max_ for XR5944.14 revealed an increase in both parameters with the dose. The inter-patient variability increased from dose level 24 mg m^−2^ onwards. Mean *C*_max_ and AUC_0–24_ were similar in the higher dose levels. This might explain the similar toxicity profile seen in the higher dose groups (from 24 mg m^−2^ onwards) and unpredictable toxicity patterns in this dose range.

The unpredictability of PKs remains a puzzle. There is a high level of protein binding with only 0.8% of unbound free drug in humans at concentrations of XR5944.14 of 100, 500 and 2500 ng ml^−1^. Serum albumin was within normal limits at study entry in all our patients. The very low metabolic turnover of [^14^C]XR5944.14 by human liver microsomes suggests that clinically significant *in vivo* interactions between XR5944.14 and coadministered drugs which are CYP enzyme inducers or inhibitors, are unlikely. Although high concentrations of XR5944.14 showed inhibition of CYPA1/2 and CYP3A4/5, these effects occurred at concentrations of 10 and 100 *μ*M, compared with the highest *C*_max_ seen in our study of 3679 ng ml^−1^. It is possible that polymorphisms in drug transporter proteins contributed to the variation in toxicity seen. The lack of correlation between toxicity and PK values means that it is difficult to recommend a dose for further study in phase II trials (Table 1). More work is needed to explain the intra- and inter-individual variation in drug handling.

## Figures and Tables

**Figure 1:**
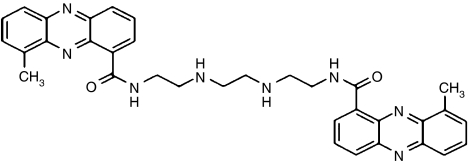
Molecular structure of XR5944.14.

**Table 1:** Dose-limiting toxicities, *C*_max_, AUC_0–∞_ and action taken

**Patient**	**DLT**	**CTC grade**	**Dose (mg m^−2^)**	**Cycle**	***C*_max_(ng ml^−1^)**	**AUC_0–**∞**_ (ng h ml^−1^)**	**Action**
1	Mucositis	3	24	2	811.78	1010	Dose reduction to 14 mg m^−2^ after cycle 2
2	Peripheral oedema	2	30	2	3679.84	4280	None
3	Acute renal failure	4	30	1	1782.83	1890	Stopped after cycle 1
4	Acute renal failure	2	30	1	1172.54	1750	Stopped after cycle 1
5	Mucositis	2	33	1	1052.93	1120	Dose reduction to 24 mg m^−2^ after cycle 1,
							stopped after cycle 2
6	Mucositis	2	36	2	2893.06	2220	Dose reduction to 24 mg m^−2^ after cycle 2
7	Mucositis	3	36	1	1966.00	1630	Dose reduction to 24 mg m^−2^ with dose-limiting mucositis again
	Neutropenic sepsis	4					
8	Mucositis	2	36	1	1799.78	1730	Dose reduction to 30 mg m^−2^ after cycle 1
9	Mucositis	2	36	1	1070.35	1250	Stopped after cycle 1
	Colitis	3					
	Renal failure	3					

**Table 2:** Worst toxicity per patient according to NCI-CTC version 2.0

		**Hb**					**Neu**					**WCC**					**Plt**					**Nausea**					**Vomiting**					**Fatigue**					**Diarrhoea**					**Mucositis**					
**Dose (mg m^−2^)**	***N***	**0**	**1**	**2**	**3**	**4**	**0**	**1**	**2**	**3**	**4**	**0**	**1**	**2**	**3**	**4**	**0**	**1**	**2**	**3**	**4**	**0**	**1**	**2**	**3**	**4**	**0**	**1**	**2**	**3**	**4**	**0**	**1**	**2**	**3**	**4**	**0**	**1**	**2**	**3**	**4**	**0**	**1**	**2**	**3**	**4**	**DLT**
3.6	2	0	1	1	0	0	2	0	0	0	0	2	0	0	0	0	2	0	0	0	0	1	1	0	0	0	1	0	1	0	0	1	1	0	0	0	2	0	0	0	0	2	0	0	0	0	0
7.2	2	0	1	1	0	0	2	0	0	0	0	2	0	0	0	0	2	0	0	0	0	1	0	0	1	0	1	0	0	1	0	2	0	0	0	0	2	0	0	0	0	2	0	0	0	0	0
14	2	0	1	1	0	0	1	1	0	0	0	1	1	0	0	0	2	0	0	0	0	0	0	1	0	0	1	0	1	0	0	0	1	1	0	0	1	1	0	0	0	2	0	0	0	0	0
24	3	0	1	2	0	0	2	0	0	1	0	2	1	0	0	0	2	1	0	0	0	0	0	2	0	0	1	1	0	1	0	2	0	1	0	0	2	0	2	0	0	2	0	0	1	0	1
30	5	0	4	1	0	0	4	0	0	1	0	4	1	0	0	0	5	0	0	0	0	3	1	1	0	0	3	2	0	0	0	4	1	0	0	0	3	1	1	0	0	3	1	1	0	0	3
33	6	0	3	3	0	0	4	1	0	1	0	4	0	2	0	0	4	2	0	0	0	4	1	1	0	0	5	0	1	0	0	3	1	2	0	0	1	5	0	0	0	1	5	0	0	0	1
36	7	1	4	2	0	0	2	0	0	4	1	2	0	3	2	0	4	3	0	0	0	3	3	1	0	0	3	4	0	0	0	3	2	1	1	0	2	2	2	1	0	1	1	4	1	0	4

DLT=dose-limiting toxicity; Hb=haemoglobin; Neu=neutrophil; Plt=platelet; WCC=total white cell count.

**Table 3:** Mean values of the pharmacokinetic parameters

**Dose level (mg m^−**2**^)**							
**Parameter**	**3.6**	**7.2**	**14**	**24**	**30**	**33**	**36**
*C*_max_ (ng ml^−1^)	104.83	268.15	309.21	1300.91	1921.20	1767.86	1951.60
*T*_max_ (h)	0.25	0.25	0.5	0.25^a^	0.333^a^	0.333^a^	0.333^a^
AUC_0–*t*_ (ng h ml^−1^)	105	221	342	987	2050	1780	1780
AUC_0–∞_ (ng h ml^−1^)	120	247	374	1050	2160	1740	1870
*λ*_*z*_ (h^−1^)	0.0118	0.0109	0.0115	0.0136	0.0162	0.0111	0.0140
*T*_½_ (h)	60.6	65.0	61.0	51.1	42.8	62.7	49.6
CL (l h^−1^ m^−2^)	30.0	29.2	37.7	23.3	16.5	20.2	19.4
MRT (h)	37.0	33.0	29.8	25.5	25.0	32.6	21.6
*V*_*z*_ (l m^−2^)	2610	2740	3300	1900	1240	2040	1440
*V*_ss_ (l m^−2^)	1090	962	1110	611	428	712	421

AUC= area under the concentration–time curve; *C*_max_=peak plasma level; CL=total plasma clearance; MRT=mean residence time; *T*_max_=time to maximal concentration; *t*_½_=terminal half-life; *λ_z_*=terminal rate constant; *V*_ss_=apparent volume of distribution at steady state; *V_z_*=apparent volume of distribution during the terminal phase.

a Median.

**Table 4:** Relationship between *C*_max_, AUC_0–*t*_, AUC_0–∞_ and dose level

**Dose level (mg m^−2^)**	**Dose level ratio**	***C*_max_ ratio**	**AUC_0–*t*_ ratio**	**AUC**_**0–**∞_ **ratio**
3.6	1	1	1	1
7.2	2.0	2.6	2.1	2.1
14	3.9	2.9	3.3	3.1
24	6.7	12.4	9.4	8.8
30	8.3	18.3	19.5	18.0
33	9.2	16.9	17.0	14.5
36	10.0	18.6	17.0	15.6
